# A Patient-Controlled Intravenous Analgesia With Tramadol Ameliorates Postpartum Depression in High-Risk Woman After Cesarean Section: A Randomized Controlled Trial

**DOI:** 10.3389/fmed.2021.679159

**Published:** 2021-05-27

**Authors:** Zhuoxi Wu, Peng Zhao, Jing Peng, Liang Fang, Jinping Ding, Guangming Yan, Yang Wang, Jing Zhu, Dongting Wang, Yang Li, Zhengqiong Chen, Qingling Zhang, Qiangting Deng, Guangyou Duan, Zhiyi Zuo, Hong Li

**Affiliations:** ^1^Department of Anesthesiology, Second Affiliated Hospital of Army Medical University, People's Liberation Army of China (PLA), Chongqing, China; ^2^Department of Anesthesiology, Chinese People's Liberation Army of China (PLA) No. 964 Hospital, Changchun, China; ^3^Department of Obstetrics, Second Affiliated Hospital of Army Medical University, People's Liberation Army of China (PLA), Chongqing, China; ^4^Department of Psychology, Second Affiliated Hospital of Army Medical University, People's Liberation Army of China (PLA), Chongqing, China; ^5^Editorial Office of Journal of Third Military Medical University, Army Medical University, People's Liberation Army of China (PLA), Chongqing, China; ^6^Department of Anesthesiology, University of Virginia, Charlottesville, VA, United States

**Keywords:** tramadol, postpartum depression, cesarean section, PCIA, perioperative period tramadol PCIA ameliorates PPD 2

## Abstract

**Background:** Postpartum depression (PPD) is a severe psychiatric disorder. Its risk is associated with the cesarean section (CS). Currently, there are few early intervention strategies for these women with PPD who underwent CS.

**Methods:** This was a parallel-group randomized controlled trial of singleton pregnant women who underwent elective CS in a tertiary referral hospital in China from October, 2017 to September, 2019. After operation, patients received randomly tramadol patient-controlled intravenous analgesia (PCIA; 4 mg/ml; TRA group), hydromorphone PCIA (0.04 mg/ml; HYD group), or ropivacaine patient-controlled epidural analgesia (PCEA; 1.5 mg/ml; ROP group) for 48 h in a 1:1:1 ratio. Total blinding during hospitalization was not feasible due to differences between the PCEA and PCIA treatments. All investigators who performed the follow-up were blinded to the group assignment.

**Outcomes:** A total of 1,230 patients were enrolled for eligibility. Intention-to-treat analysis showed reduced incidence of PPD in the TRA group (*n* = 27 [6.6%]) than that in the HYD (10.2%, OR 1.62, 95% CI 0.98~2.68; *p* = 0.059) and ROP groups (10.5%, OR 1.66, 95% CI 1.01~2.75; *p* = 0.046) at 4 weeks post-operation, however, the difference was not statistically significant (Bonferroni corrected *p* = 0.118, *p* = 0.098, respectively). Subgroup analysis in high-risk women (preoperative Edinburgh Postpartum Depression Scale [EPDS] ≥10) showed a significantly lower incidence of PPD in the TRA group (16.5%) than in the HYD (32.6%) and ROP groups (30.9%) (Bonferroni corrected *p* = 0.022 and *p* = 0.038, respectively). The per-protocol analysis yielded similar results. Reported adverse events (AEs) were mostly mild. None of the women or infant discontinued treatment due to AEs.

**Conclusions:** Tramadol PCIA after CS in high-risk women can help to reduce the risk of PPD at 4 weeks after elective CS.

**Clinical Trial Registration:** https://clinicaltrials.gov/ct2/show/NCT03309163?term=ETPPD&draw=2&rank=1; ClinicalTrials.gov (NCT03309163).

## Introduction

Postpartum depression (PPD) is a severe psychiatric disorder that occurs during the perinatal period or within 4 weeks after delivery (1). Its prevalence in developed countries is ~10% (2) and has reached 11.8–15.7% in China (3–5). The cesarean delivery rate in China was 36.7% in 2018 (6), which is more than twice above the recommended level (7). Studies have suggested that cesarean delivery was associated with the risk of PPD (8, 9). Without intervention, PPD may persist up to 2 years beyond the postpartum period and ~40% of affected women will have a relapse in subsequent pregnancies (10).

Common treatments for PPD include pharmacological and psychological therapy. However, a study reported that the use of antidepressants, especially selective serotonin reuptake inhibitors (SSRIs), during pregnancy represented differences in teratogenic activity to the infants (11). Hence, most women are unwilling to receive antidepressants during the perinatal period. Physicians may also be hesitant to prescribe antidepressants (12) or prescribe insufficient doses in consideration of fetal and infant exposure to the medications (13). Recently, a novel antidepressant has been shown to relieve PPD (14). However, it was only effective after the onset of PPD. Although, psychological therapies may be as effective as early interventions for PPD, they have limited popularity and acceptability among patients (15), especially in developing countries. Furthermore, patients usually seek medical attention only when the symptoms become severe. These challenges hinder women from receiving timely and proper treatment during the perinatal period.

Postoperative pain is a risk factor for PPD (16). Patient-controlled intravenous analgesia (PCIA) is one of the most commonly used methods for postoperative pain management (17). Tramadol, a commonly used medication after CS in China, is a weak μ-opioid receptor agonist that is effective and well-tolerated for relieving postoperative pain (18, 19). It also inhibits serotonin and norepinephrine uptake, similar to tricyclic antidepressants (20–22). In addition, only ~0.1% of tramadol and its metabolites are detected in the breast milk (18), and the relative infant doses are within the safety level for breastfeeding (23, 24). Tramadol has been shown to have antidepressant-like effects in animal studies (25–27). Our pilot trial (19) found that tramadol PCIA following CS reduced the incidence of depression in the early postpartum period. However, there is limited research on early intervention with tramadol to prevent PPD.

We hypothesize that PCIA with tramadol after CS would be reduce the risk of PPD, which may be a rational treatment option for patients facing the dual challenge of PPD and postoperative pain. We conducted a randomized clinical trial (RCT) to examine the effects of tramadol PCIA vs. hydromorphone PCIA and ropivacaine patient-controlled epidural analgesia (PCEA) on PPD in women who underwent CS.

## Materials and Methods

### Trial Design

**Figure 1:**
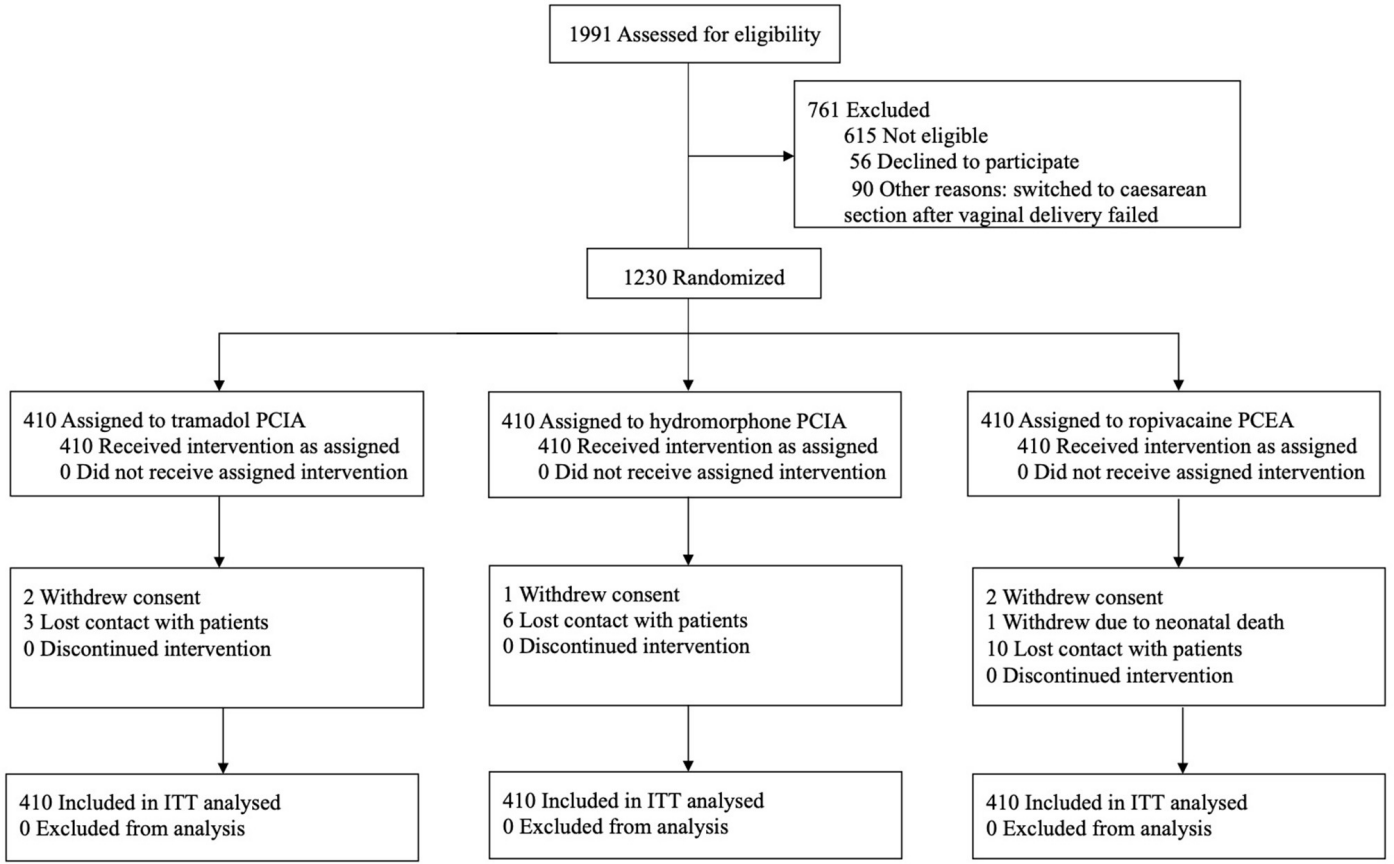
Trial flow ITT analyses included all randomized patients in the groups to which they were randomly assigned. A total of 25 patients (2%) (5 in the TRA group, 7 in the HYD group, and 13 in the ROP group) lacked primary outcome data. ITT, Intention-To-Treat, PCIA, Patient-controlled intravenous analgesia, PCEA, Patient-controlled epidural analgesia.

This was a prospective, parallel-group RCT of Chinese patients who underwent elective CS in a tertiary referral hospital (The Second Affiliated Hospital of the Army Medical University) in Chongqing, China between 15 October 2017 and 5 September 2019 (Figure 1). The study design and methods were previously published (28). The study protocol was approved by the Medical Ethics Committee of the Second Affiliated Hospital, Army Military Medical University (Approval ID: 2017–026) and registered with ClinicalTrials.gov (Identifier NCT03309163).

### Participants

Written informed consent was obtained from all patients in the ward after admission to the hospital and prior to the initiation of any research procedures. All participants were the singleton full-term pregnant women in an aged 20–40 years. Inclusion criteria were aged 20–40 years; American Society of Anesthesiologists class II; singleton full-term pregnant women who received elective CS (eCS) and voluntary postoperative controlled analgesia; and consented to participate in the study. Exclusion criteria included history of diagnosed mental disorders, prior use of psychiatric medication or psychotropic substances; history of neurological diseases such as epilepsy; history of previously diagnosed depression; with suicidal ideations or history of suicide; history of drug, alcohol or opioid abuse; received monoamine oxidase inhibitor treatment at present or in the past 14 days; participated in other clinical studies; with severe heart disease, brain disease, liver disease, or kidney disease; with allergies to tramadol or opioids; with any contraindication to combined spinal-epidural anesthesia (CSEA); and inability to communication or cooperate.

At the time of preoperative recruitment, patients were screened and eligible patients were recruited into the study and the study procedures were explained to them before beginning the study. Patients were assessed by professionally trained researchers using the patient self-report Edinburgh Postpartum Depression Scale (EPDS), a commonly used instrument for screening perinatal depression (29, 30), and Generalized Anxiety Disorder 7-item (GAD-7) questionnaire, a common tool for screening GAD in general hospitals in China (GAD-7 score > 9 indicates the presence of GAD) (31, 32). All eligible participants were monitored according to routine practice after entering the operating room. Then, a standardized CS under standard CSEA was carried out for each patient by an experienced anesthesiologist and obstetrician. After the operation, the participants received PCIA or PCEA immediately and were monitored in the obstetrics intensive care unit for 6 h before returning to the general ward.

### Randomization and Blinding

Depression during pregnancy is a critical risk factor for PPD (33, 34). Therefore, an preoperative EPDS ≥ 10, a recommended cut-off score for screening depressive illness in the Chinese general postnatal population (30), was used as the stratification factor in the randomization process. And we defined these women with preoperative EPDS > 10 as a high-risk group. We used a computer-generated randomization sequence to randomly allocate patients in a 1:1:1 ratio into three analgesia groups by stratified block randomization.

Although, it is not feasible to completely blind patients or investigators during operation and hospitalization due to the apparent difference between PCEA and PCIA, the investigators who conducted postoperative and long-term follow-ups were blinded to the group assignment. Participants, care providers, and investigators were blinded to the assignment of the two PCIA groups. Moreover, statisticians were blinded to the group assignment until the analysis of the research outcomes was completed.

### Interventions

A primary control group (hydromorphone [HYD] PCIA, Renfu, China, 0.04 mg/ml) and a second control group (ropivacaine [ROP] PCEA, AstraZeneca, Sweden, 1.5 mg/ml) were set up in parallel to the experimental group (tramadol [TRA] PCIA, Gran tai, Germany, 4 mg/ml with a maximum daily dose of 400 mg). The above three treatments were diluted in 0.9% saline. The patient-controlled analgesia (PCA) pumps were programmed to administer a background infusion at 4.0 ml/h, with PCIA dose of 1 ml, PCEA dose of 4 ml, lockout period of 15 min, and maintenance duration of 48 h. Immediately after the operation, the PCA medications were administered for 48 h. When inadequate analgesia occurred (resting pain visual analog score [VAS] > 40) (35), patients were given a time-parameter setting adjustment for the PCA pump or were supplemented with the medications in the respective groups until the VAS score was ≤ 30.

### Outcomes

#### Primary Outcome

The primary outcome was the incidence of PPD at 4 weeks after eCS. assessed using the EPDS, and diagnosed with the American Psychiatric Association's Diagnostic and Statistical Manual of Mental Disorders, Fifth Edition (DSM-5) (1) by a professional psychiatrist.

#### Secondary Outcomes

Secondary outcomes included the incidence of PPD at 3 months after eCS; the incidence of anxiety state (as assessed by GAD-7) at 4 weeks and 3 months after the CS; the quality of recovery (as assessed by QoR-15; higher score indicates better quality of maternal recovery) at 48 h post operation; pain at rest [assessed by numerical rating scale (36) (NRS; 0 = no pain, 10 = unbearable pain)] and Ramsay sedation score (37) at 6, 12, 24, and 48 h post-operation; the incidence of inadequate analgesia (VAS > 40) at 48 h post operation; early walking time (determined by the time point when patients could ambulate independently); lactation onset time (based on patient self-reported onset time); sleep quality (self-reported by patients and rated as “very poor to poor,” “general,” or “good to very good”) on day 0 and day 1 post-operation; incidence of related postoperative adverse events (AEs) including nausea and vomiting, dizziness, pruritus, headache, and hypoesthesia; the length of hospital stay post-operation and hospitalization costs. All of the data were collected in the phases of postoperative follow-up during the hospitalization period or long-term follow-up after discharge except for AEs which were monitored and recorded post-operation until discharge.

QoR-15 is an easy-to-use score for assessing the quality of postoperative recovery from the patient's perspective. It has good validity, reliability, and reproducibility in patients who have undergone surgery under anesthesia (38, 39). The QoR-15 includes two parts—physical and mental well-being, comprising items from five dimensions (pain, physical comfort, physical independence, psychological support, and emotional state).

### Procedures

The study had four phases: preoperative recruitment, operation under anesthesia, postoperative follow-up during the hospitalization period, and long-term follow-up after discharge.

At the time of preoperative recruitment, participants were screened and recruited in the obstetric ward after admission and before eCS. Information about the demographic characteristics was recorded. The participants were assessed by professionally trained researchers on the EPDS and the GAD-7. Considering the stratification factor (preoperative EPDS ≥ 10), eligible subjects were randomly assigned to receive tramadol PCIA, hydromorphone PCIA, or ropivacaine PCEA. During operation under anesthesia, subjects were routinely monitored after entering the operating room. Then, a standardized CS under standard CSEA was implemented for each subject by an experienced anesthesiologist and obstetrician. After the operation, the patients from the venous analgesia group had the epidural catheter removed and was connected to the PCIA pump, but the epidural catheter of the PCEA group was retained. All patients received PCIA or PCEA immediately post-operation, were trained and supervised in the obstetrics intensive care unit for 6 h, and then returned to the general ward. Outcome data were collected during the postoperative follow-up in the hospital by a specialized researcher. Long-term follow-up after discharge was performed by a professionally trained researcher at 4 weeks (±3 days) and 3 months (±7 days) postpartum *via* telephone interviews. The specific content of the phone interview was as follows: (1) ask two emotionally related questions: (a) during the past month, have you often been bothered by feeling down, depressed, or hopeless? (b) have you often been bothered by little interest or pleasure in doing things? (2) ask patients if they completed the self-reported EPDS and GAD-7 questionnaires as they did during hospitalization before the CS. The EPDS and GAD-7 questionnaires were sent *via* “WeChat” (the most commonly used social networking software in China). Participants who were assessed to be at risk of depression (EPDS ≥ 10) were referred to a professional psychiatrist for further clinical assessment by telephone or face-to-face consultation using the DSM-5 to determine if patients had PPD. An appropriate treatment plan for PPD was developed at the psychiatrist's discretion, taking into account the severity of the patient's symptoms, and her functional status, including their ability to care for and relate to the new-born (40). Participants who were uncontactable for more than 1 week were considered lost to follow-up. Finally, data analysis is performed by specialized statisticians.

A phone and WeChat return visit to the last randomized participant of the study was completed on Sep 5, 2019.

### Statistical Analysis

We estimated the incidence of PPD to be ~15% based on a previous study that showed that the incidence of PPD after CS in Chinese women is ~7.3 to 26.3% (41). Considering that the postoperative Hospital Anxiety and Depression Scale scores in the tramadol group were less than half of those in the hydromorphone group at 1 week post-operation in our previous pilot trial (19), we hypothesized that the risk of PPD would be reduced by 50% in the TRA group compared with other control groups, that is, an estimated incidence of 7.5% in the TRA group and 15% in the control group. Based on a significance level of 0.05, power of 0.9, and considering an ~10% loss to follow-up, we planned to enroll 410 patients for each group, with a total sample size of 1,230 patients.

We analyzed the outcome data with the intention-to-treat (ITT) principal and repeated the analysis for the primary endpoint with the per-protocol (PP) principal. The ITT analyses included all randomized patients in each group. For patients who withdrew from the study, the data collected at the point of withdrawal were used as part of the ITT analysis. Patients who failed to follow the study protocol, lacked data for the primary outcome, or presented severe complications during CS or hospitalization were excluded from the PP analysis. We did not perform an interim analysis. Categorical variables were compared between groups using the χ2 test, continuity correction χ2 test, or Fisher's exact test. Continuous variables were compared between groups using analysis of *t*-test, variance (ANOVA) with *post-hoc* least significant difference test, or Mann-Whitney *U*-test. The difference (and 95% CI for the difference) between the two medians was calculated using the Hodges-Lehmann estimator, and the rate difference with 95% CI between different groups was also calculated. Variables were summarized as mean ± standard deviation (SD), number (frequency), and median (interquartile range [IQR]). Statistical analyses were performed using SPSS software (version 24.0, SPSS, Chicago, IL, USA), with two-tailed *p* < 0.05 being considered statistically significant. *P*-values were adjusted using Bonferroni correction of 0.05/2. Major outcomes were compared between the TRA group and HYD group and the TRA group and ROP group. Because prenatal depression may affect the primary outcome, subgroup analyses in women with preoperative EPDS ≥ 10 (high risk women) were also performed.

An exploratory stepwise binary logistic regression analysis using the forward logistic regression model was performed to investigate whether the onset of PPD at 4 weeks after CS was affected by the selected demographic and clinical characteristics. According to the model selection principle, the model with the smallest Akaike Information Criterion (AIC) value was selected (42). The independent variables included age, BMI, gestational weeks, complication, number of CSs, history of surgery (except CS), sleep quality in the last week, preoperative EPDS and GAD-7 scores, operation duration, postpartum hemorrhage, occupation, educational level, spouse's occupation, spouse's educational level, marital status, marital relationship, monthly household income, QoR-15, NRS at 6, 12, 24, and 48 h after operation, early walking time, lactation onset time, sleep quality on the day 0 and day 1 post-operation, the length of hospital stay post-operation, and hospitalization costs.

## Results

Of the 1,230 patients who were enrolled in the study, 410 were randomized to receive tramadol PCIA, hydromorphone PCIA, or ropivacaine PCEA, respectively (Figure 1). At 4 weeks after the operation, 25 patients (2%) lacked data for the primary outcome (six withdrew during hospitalization, 19 were lost to follow-up). Fifty-six patients (4.6%) lacked data on the incidence of PPD at 3 months. The characteristics of the randomized groups and subgroups are shown in Table 1 and Supplementary Table 1, respectively.

**Table 1:** Demographics and perioperative clinical characteristics.

	**Tramadol** ** PCIA** ***n* = 410**	**Hydromorphone** ** PCIA** ***n* = 410**	***P-*value**	**Tramadol** ** PCIA** ***n* = 410**	**Ropivacaine** ** PCEA** ***n* = 410**	***P-*value**
Age (years)^ad^	30.8 ± 4.1	31.2 ± 4.2	0.20	30.8 ± 4.1	31 ± 4.4	0.70
BMI (kg/m^2^)^ad^	27.7 ± 3.8	27.8 ± 3.3	0.89	27.7 ± 3.8	27.7 ± 3.0	0.78
Gestational weeks^be^			0.35			0.26
Pre-term	49 (12.0%)	59 (14.4%)		49 (12.0%)	38 (9.3%)	
Full-term	361 (88.0%)	351 (85.6%)		361 (88.0%)	371 (90.5%)	
Post-term	0 (0%)	0 (0%)		0 (0%)	1 (0.2%)	
Complication^be^			0.89			0.25
No	157 (38.3%)	160 (39.0%)		157 (38.3%)	174 (42.4%)	
Yes	253 (61.7%)	250 (61.0%)		253 (61.7%)	236 (57.6%)	
Number of CSs^be^			0.36			0.78
0	129 (31.5%)	120 (29.3%)		129 (31.5%)	139 (33.9%)	
1	260 (63.4%)	277 (67.6%)		260 (63.4%)	248 (60.5%)	
2	20 (4.9%)	12 (2.9%)		20 (4.9%)	21 (5.1%)	
3	1 (0.2%)	1 (0.2%)		1 (0.2%)	2 (0.5%)	
History of surgery (other than CS)^be^			0.43			0.52
0	330 (80.5%)	314 (76.6%)		330 (80.5%)	322 (78.5%)	
1	73 (17.8%)	87 (21.2%)		73 (17.8%)	76 (18.5%)	
2	7 (1.7%)	7 (1.7%)		7 (1.7%)	10 (2.4%)	
3	0 (0%)	1 (0.2%)		0 (0%)	2 (0.5%)	
4	0 (0%)	1 (0.2%)		0 (0%)	0 (0%)	
Sleep quality in the last week^be^			0.71			0.96
Very poor to poor	95 (23.2%)	97 (23.7%)		95 (23.2%)	94 (22.9%)	
General	176 (42.9%)	185 (45.1%)		176 (42.9%)	180 (43.9%)	
Good to very good	139 (33.9%)	128 (31.2%)		139 (33.9%)	136 (33.2%)	
Preoperative EPDS^be^			0.50			0.93
<10	313 (76.3%)	322 (78.5%)		313 (76.3%)	315 (76.8%)	
≥10	97 (23.7%)	88 (21.5%)		97 (23.7%)	95 (23.2%)	
Preoperative GAD-7^be^			0.82			>0.99
≤ 9	399 (97.3%)	401 (97.8%)		399 (97.3%)	400 (97.6%)	
>9	11 (2.7%)	9 (2.2%)		11 (2.7%)	10 (2.4%)	
Operation duration (mins)^ad^	84.03 ± 23.94	83.92 ± 30.18	0.95	84.03 ± 23.94	82.62 ± 25.74	0.42
Postpartum hemorrhage^be^			0.17			0.52
No	397 (96.8%)	388 (94.6%)		397 (96.8%)	398 (97.1%)	
Yes	13 (3.2%)	22 (5.4%)		13 (3.2%)	12 (2.9%)	
PCIA consumption (mL)^cf^	178(155,213)	182(154,210)	0.74	178(155,213)	182(152,210)	0.74
Occupation^be^			0.20			0.13
Peasant	6 (1.5%)	11 (2.7%)		6 (1.5%)	13 (3.2%)	
Worker	7 (1.7%)	13 (3.2%)		7 (1.7%)	8 (2.0%)	
Student	1 (0.2%)	0 (0%)		1 (0.2%)	0 (0%)	
Soldier	0 (0%)	2 (0.5%)		0 (0%)	0 (0%)	
Office clerk or civil servant	160 (39.0%)	145 (35.4%)		160 (39.0%)	134 (32.7%)	
Other	236 (57.6%)	239 (58.3%)		236 (57.6%)	255 (62.2%)	
Educational level^be^			0.28			0.49
≤ 9 years	89 (21.7%)	72 (17.6%)		89 (21.7%)	76 (18.5%)	
10~12 years	83 (20.2%)	94 (22.9%)		83 (20.2%)	91 (22.2%)	
>12 years	238 (58.0%)	244 (59.5%)		238 (58.0%)	243 (59.3%)	
Spouse's occupation^be^			0.13			0.28
Peasant	8 (2.0%)	9 (2.3%)		8 (2.0%)	14 (3.5%)	
Worker	12 (2.9%)	28 (6.8%)		12 (2.9%)	16 (3.9%)	
Student	0 (0%)	0 (0%)		0 (0%)	0 (0%)	
Soldier	5 (1.2%)	5 (1.2%)		5 (1.2%)	7 (1.7%)	
Office clerk or civil servant	142 (34.6%)	130 (31.7%)		142 (34.6%)	118 (28.8%)	
Other	243 (58.3%)	238 (58.0%)		243 (58.3%)	255 (63.3%)	
Spouse's educational level^be^			0.46			0.50
≤ 9 years	68 (16.6%)	56 (13.7%)		68 (16.6%)	57 (13.9%)	
10~12 years	106 (25.9%)	115 (28.0%)		106 (25.9%)	116 (28.3%)	
>12 years	236 (57.6%)	239 (58.3%)		236 (57.6%)	237 (57.8%)	
Marital status^be^			0.22			0.19
Married	404 (98.5%)	408 (99.5%)		404 (98.5%)	409 (99.8%)	
Unmarried	3 (0.7%)	2 (0.5%)		3 (0.7%)	0 (0%)	
Divorced	3 (0.7%)	0 (0%)		3 (0.7%)	1 (0.2%)	
Monthly household income (Yuan)^be^			0.90			0.20
0–1500	1 (0.2%)	2 (0.5%)		1 (0.2%)	2 (0.5%)	
1500–4500	43 (10.5%)	43 (10.5%)		43 (10.5%)	55 (13.4%)	
4500–9000	236 (57.6%)	233 (56.8%)		236 (57.6%)	227 (55.4%)	
9000–35000	129 (31.5%)	129 (31.5%)		129 (31.5%)	120 (29.3%)	
>35000	1 (0.2%)	3 (0.7%)		1 (0.2%)	6 (1.5%)	
Marital relationship^be^			0.12			0.10
Bad	1 (0.2%)	4 (1.0%)		1 (0.2%)	7 (1.7%)	
General	14 (3.4%)	6 (1.5%)		14 (3.4%)	11 (2.7%)	
Good	395 (96.3%)	400 (97.6%)		395 (96.3%)	392 (95.6%)	

*Data are described as*

a *mean ± standard deviation*,

b *number (percentage)*

c *median with Iinterquartile range*.

d *Analyzed by the Independent-Samples T-Test*.

e *Analyzed by the Chi-square test*.

f *Analyzed by the Mann-Whitney U-test*.

*BMI, Body mass index; EPDS, Edinburgh Postpartum Depression Scale; GAD, Generalized Anxiety Disorder; PCEA, Patient-controlled epidural analgesia; PCIA, Patient-controlled intravenous analgesia; CS, cesarean section*.

### Primary Outcome

**Figure 2:**
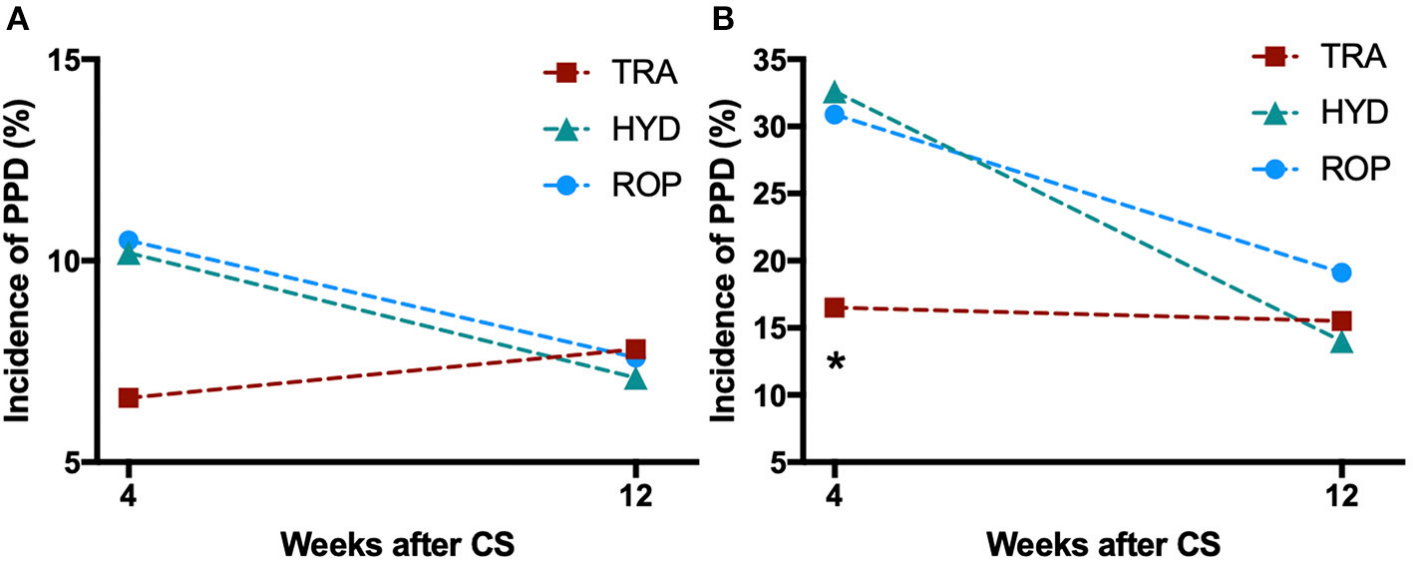
Changes in the incidence of PPD from 4 weeks to 3 months after CS in **(A)** all patients; and **(B)** patients at high risk for PPD. *P*-values were calculated by the χ2 test and adjusted by Bonferroni correction. *p < 0.05 vs. HYD and ROP groups. PPD, Postpartum depression; TRA, Tramadol PCIA; HYD, Hydromorphone PCIA; ROP, Ropivacaine PCEA; CS, cesarean section.

PPD occurred in 112 (9.3%) of the 1,205 patients at 4 weeks after CS. In the ITT analysis (Table 2 and Figure 2A), the incidence of PPD in the TRA group (6.6% [27/410]) was not statistically different from that in the HYD group (10.2% [42/410]), but was significantly lower than that in the ROP group (10.5% [43/410]). After Bonferroni correction, these differences were not statistically significant. Similarly, PP analysis showed that the incidence of PPD in the TRA group (6.7% [27/405]) was lower than that in the HYD group (10.4% [42/403], OR 1.63, 95% CI: 0.98~2.70) and the ROP group (10.8% [43/397], OR 1.70, 95% CI 1.03~2.81). However, the differences were not statistically significant (*p* = 0.056 [Bonferroni corrected *p* = 0.112]; *p* = 0.037 [Bonferroni corrected *p* = 0.074]).

**Table 2:** Primary and secondary outcomes of tramadol group compared to control groups.

**Primary control**	**Tramadol PCIA** ***n* = 410**	**Hydromorphone PCIA** ***n* = 410**	**OR or difference** ** (95% CI)**	***P*-value**	**Bonferroni correction**
**Primary outcome**					
Incidence of PPD at the 4th week^be^	27 (6.6%)	42 (10.2%)	OR = 1.62 (0.98~2.68)	0.059	0.118
**Secondary outcomes**					
Incidence of GAD at the 4th week^be^	14 (3.4%)	16 (3.9%)	OR = 1.15 (0.55~2.38)	0.710	>0.99
Incidence of PPD at the 3rd month^be^	32 (7.8%)	29 (7.1%)	OR = 0.90 (0.53~1.52)	0.690	>0.99
Incidence of GAD at the 3rd month^be^	10 (2.4%)	14 (3.4%)	OR = 1.41 (0.62~3.22)	0.407	0.814
QoR-15 at 48 h after operation^ad^	121.30 ± 15.75	118.06 ± 17.56	D = 3.24 (0.43~6.05)	**0.018**	**0.036**
NRS for pain at rest (score)^cf^					
6 h after operation	4 (2, 6)	4 (2, 6)	D = 0 (0~1)	0.145	0.29
12 h after operation	3 (2, 4)	3 (2, 4)	D = 0 (0~0)	0.731	>0.99
24 h after operation	2 (1, 4)	3 (2, 4)	D = 0 (0~0)	0.066	0.132
48 h after operation	2 (0, 3)	2 (0, 3)	D = 0 (0~0)	0.264	0.528
Inadequate analgesia after operation^be^	31 (7.6%)	42 (10.2%)	OR = 1.40 (0.86~2.27)	0.177	0.354
Ramsay sedation score^cf^	2 (2, 2)	2 (2, 2)	D = 0 (0~0)	0.576	>0.99
Early walking time^ad^ (h)	25.68 ± 7.65	26.09 ± 7.54	D = −0.53 (−1.62~0.55)	0.334	0.668
Lactation onset time^ad^ (h)	27.21 ± 17.99	26.95 ± 18.08	D = 0.26 (−2.47~2.99)	0.852	>0.99
Hospital stays^ad^ (days)	3.32 ± 0.67	3.34 ± 0.70	D = −0.03 (−0.14~0.09)	0.832	>0.99
Sleep quality on the day 0 after operation^be^				0.738	>0.99
Very poor to poor	239 (58.3%)	234 (57.1%)	-		
General	83 (20.2%)	92 (22.4%)	-		
Good to very good	88 (21.5%)	84 (20.5%)	-		
Sleep quality on the day 1 after operation^be^				0.305	0.610
Very poor to poor	65 (17.0%)	78 (20.5%)	-		
General	85 (22.2%)	91 (23.9%)	-		
Good to very good	233 (60.8%)	212 (55.6%)	-		
**Secondary control**	**Tramadol PCIA** **n** **=** **410**	**Ropivacaine PCEA** **n** **=** **410**	**OR or difference** **(95% CI)**	***P*****-value**	**Bonferroni correction**
**Primary outcome**					
Incidence of PPD at the 4th week^be^	27 (6.6%)	43 (10.5%)	OR = 1.66 (1.01~2.75)	**0.046**	0.092
**Secondary outcomes**					
Incidence of GAD at the 4th week^be^	14 (3.4%)	22 (5.4%)	OR = 1.60 (0.81~3.18)	0.173	0.346
Incidence of PPD at the 3rd month^be^	32 (7.8%)	31 (7.6%)	OR = 0.97 (0.58~1.62)	0.896	>0.99
Incidence of GAD at the 3rd month^be^	10 (2.4%)	9 (2.2%)	OR = 0.90 (0.36~2.23)	0.816	>0.99
QoR-15 at 48 h after operation^ad^	121.30 ± 15.75	116.22 ± 18.20	D = 5.08 (2.19~7.96)	** <0.001**	** <0.001**
NRS for pain at rest (score)^cf^					
6 h after operation	4 (2, 6)	4 (2, 6)	D = 0 (0~0)	0.651	>0.99
12 h after operation	3 (2, 4)	4 (2, 5)	D = −1 (−1~−1)	** <0.001**	** <0.001**
24 h after operation	2 (1, 4)	3 (2, 5)	D = −1 (−1~−1)	** <0.001**	** <0.001**
48 h after operation	2 (0, 3)	2 (0, 3)	D = 0 (−1~0)	**0.001**	**0.002**
Inadequate analgesia after operation^be^	31 (7.6%)	40 (9.8%)	OR = 1.32 (0.81~2.16)	0.264	0.528
Ramsay sedation score^cf^	2 (2, 2)	2 (2, 2)	D = 0 (0~0)	0.795	>0.99
Early walking time^ad^ (h)	25.68 ± 7.65	25.94 ± 7.41	D = −0.42 (−1.52~0.68)	0.455	0.910
Lactation onset time^ad^ (h)	27.21 ± 17.99	28.35 ± 17.66	D = −1.13 (−3.88~1.62)	0.419	0.838
Hospital stay^ad^ (days)	3.32 ± 0.67	3.23 ± 0.69	D = 0.08 (−0.03~0.20)	0.188	0.376
Sleep quality on day 0 after surgery^be^				**0.009**	**0.018**
Very poor to poor	239 (58.3%)	279 (68.0%)	-		
General	83 (20.2%)	71 (17.3%)	-		
Good to very good	88 (21.5%)	60 (14.6%)	-		
Sleep quality on day 1 after surgery^be^				**0.002**	**0.004**
Very poor to poor	65 (17.0%)	102 (27.6%)	-		
General	85 (22.2%)	78 (21.1%)	-		
Good to very good	233 (60.8%)	190 (51.4%)	-		

*Data are described as*

a *mean ± standard deviation*,

c *median with interquartile range*,

b *number (percentage). 4th week and 3rd month refers to the 4th week and 3rd month after CS, respectively. Ramsay sedation score is the average score of the four time-points after operation (6, 12, 24, and 48 h)*.

d *Analyzed by the Independent-Samples T-Test*.

e *Analyzed by the Chi-square test*.

f *Analyzed by the Mann-Whitney U-test*.

*CI, Confidence interval; D, Mean difference; GAD, Generalized Anxiety Disorder; NRS, Numerical rating scale; OR, Odds ratio; QoR, Quality of recovery; PCEA, Patient-controlled epidural analgesia; PCIA, Patient-controlled intravenous analgesia; PPD, Postpartum depression. The bold values mean the significant differences (p < 0.05)*.

### Subgroup Analysis

We performed a subgroup analysis (ITT analysis) in women with preoperative EPDS ≥ 10, indicating high risk of PPD (Table 3 and Figure 2B). The incidence of PPD in the TRA group (16.5% [16/97]) was significantly lower than that in the HYD (32.6% [28/86], OR 2.44, 95% CI 1.21~4.93; *p* = 0.011) and the ROP groups (30.9% [29/94], OR 2.49, 95% CI 1.25~4.95; *p* = 0.019), which remained statistically significant even after Bonferroni correction (*p* = 0.022 and *p* = 0.038, respectively). In the PP analysis, the incidence of PPD in the TRA group (16.7% [16/96]) was significantly lower than that in the HYD group (33.3% [28/84], OR 2.50, 95% CI 1.24~5.05, *p* = 0.009; Bonferroni corrected *p* = 0.018) and the ROP group (31.1% [28/90], OR 2.26, 95% CI 1.12~4.54, *p* = 0.021; Bonferroni corrected *p* = 0.042) even after Bonferroni correction.

**Table 3:** Subgroup analysis comparing the incidence PPD in the tramadol group with those in primary and secondary control groups.

**Primary control**	**Tramadol** ** PCIA** ***n* = 97**	**Hydromorphone** ** PCIA** ***n* = 86**	**OR** ** (95% CI)**	***P-*value**	**Bonferroni correction**
Incidence of PPD at the 4th week					
Yes	16 (16.5%)	28 (32.6%)	OR = 2.44 (1.21~4.93)	**0.011**	**0.022**
No	81 (83.5%)	58 (67.4%)			
Incidence of PPD at the 3rd month					
Yes	15 (15.5%)	12 (14.0%)	OR = 0.89 (0.39~2.02)	0.774	>0.99
No	82 (84.5%)	74 (86.0%)			
**Secondary control**	**Tramadol** ** PCIA** **n** = **97**	**Ropivacaine** ** PCEA** **n** = **94**	**OR** **(95% CI)**	***P-*****value**	**Bonferroni correction**
Incidence of PPD at the 4th week					
Yes	16 (16.5%)	29 (30.9%)	OR = 2.49 (1.25~4.95)	**0.019**	**0.038**
No	81 (83.5%)	65 (69.1%)			
Incidence of PPD at the 3rd month					
Yes	15 (15.5%)	18 (19.1%)	OR = 1.30 (0.61~2.75)	0.501	>0.99
No	82 (84.5%)	76 (80.9%)			

*Data are described as number (percentage). Analyzed by the Chi-square test in women at high risk of PPD. 4th week and 3rd month refers to the 4th week and 3rd month after CS, respectively. CI, Confidence interval; PCIA, Patient-controlled intravenous analgesia; PPD, Postpartum depression; OR, Odds ratio. The bold values mean the significant differences (p < 0.05)*.

### Secondary Outcomes

**Figure 3:**
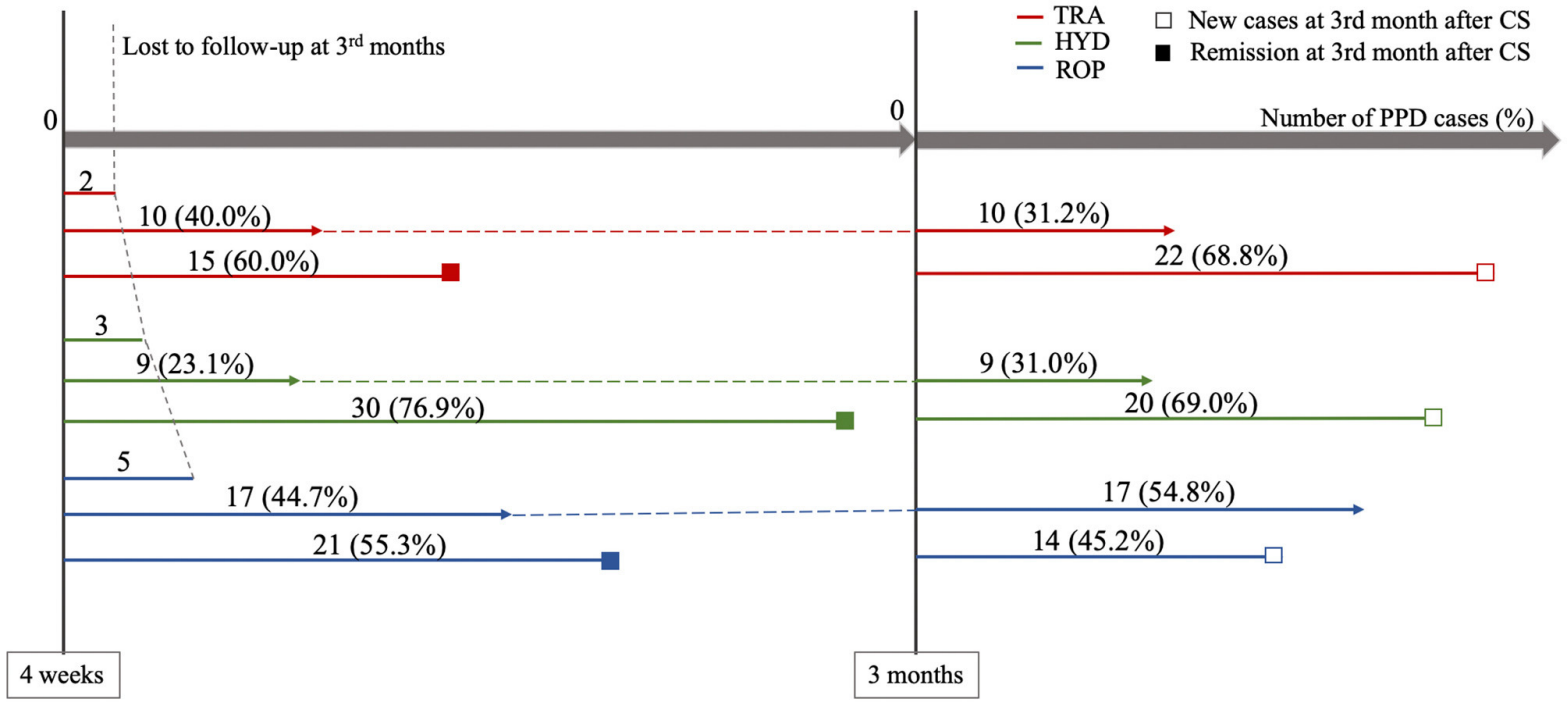
Remission rate at 3 months after CS and proportion of new cases of PPD between 4 weeks and 3 months. The red (2), green (3), and blue (5) short lines on the left of figure represented the subjects suffering from PPD at the 4 weeks after CS, which were lost to follow-up at the 3 months. The lines in the middle of three colors (10, 9, and 17) that followed by dashed lines to the next time point, represented the subjects suffering from PPD at 4 weeks and 3 months after CS. The lines at the bottom of three colors (15, 30, and 21) that marked with solid boxes, represented the subjects suffering from PPD at 4 weeks but remitted at 3 months after CS. The three lines (22, 20, and 14) on the right side of figure that marked with hollow boxes, represented new PPD cases at 3 months after CS. TRA, Tramadol PCIA; HYD, Hydromorphone PCIA; ROP, Ropivacaine PCEA; PPD, Postpartum depression; CS, cesarean section.

PPD occurred in 92 (7.9%) of the 1,170 patients at 3 months after CS. The incidence of PPD in the TRA group (7.8% [32/410]) was not statistically different from that in the HYD (7.1% [29/410]) and ROP groups (7.6% [31/410]) (Table 2 and Figure 2A). Subgroup analysis in women with a high risk of PPD also showed no differences (Table 3 and Figure 2B). The incidence of PPD at 3 months after operation was largely similar to that at 4 weeks in the TRA group whereas the incidence of PPD at 3 months declined in the control groups. To investigate the changes in the incidence of PPD between 4 weeks and 3 months post-operation, the remission rate after 3 months, and the proportion of new cases of PPD between 4 weeks and 3 months were compared among the three groups (Figure 3). At 3 months, the remission rate of cases of depressive episode at 4 weeks in the TRA group (60% [15/25]) was not statistically different from that in the HYD (76.9 [30/39], OR 2.22, 95% CI 0.75~6.63, *p* = 0.148) and ROP groups (55.3% [21/38], OR 0.82, 95% CI 0.30~2.29, *p* = 0.710). The proportion of new cases of PPD between 4 weeks and 3 months in the TRA group (68.8% [22/32]) was also not statistically different from that in the HYD (69.0% [20/29], OR 1.01, 95% CI 0.34~2.99, *p* = 0.986) and ROP groups (45.2% [14/31], OR 0.37, 95% CI 0.13~1.05, *p* = 0.059).

Other secondary outcomes are presented in Table 2. The mean ± SD QoR-15 score at 48 h after CS in the TRA group (121.30 ± 15.75) was significantly higher than that in the HYD (118.06 ± 17.56; Difference [D] = 3.24, 95% CI 0.43~6.05; *p* = 0.018) and the ROP groups (116.22 ± 18.20; D=5.08, 95% CI 2.19~7.96; *p* < 0.001). After Bonferroni correction, the difference was still statistically significant (*p* = 0.036 and *p* < 0.001, respectively). Sleep quality on day 0 or day 1 post-operation in the TRA group was not different from that in the HYD group (*p* = 0.738 and *p* = 0.305, respectively) but significantly better than the ROP group (*p* = 0.009 and *p* = 0.002, respectively, Bonferroni corrected *p* = 0.018 and *p* = 0.004, respectively).

The median (IQR) NRS scores at 6, 12, 24, and 48 h post-operation in the TRA group (4[2–6], 3[2–4], 2[1–4], and 2[0–3], respectively) were not significantly different from the HYD group (4[2–6], 3[2–4], 3[2–4], and 2[0–3], respectively; *p* = 0.145, *p* = 0.731, *p* = 0.066, and *p* = 0.264, respectively). The median (IQR) NRS scores at 12, 24, and 48 h post-operation in the TRA group (3[2–4], 2[1–4], and 2[0–3], respectively) were significantly lower than that in the ROP group (4[2–5], 3[2–5], and 2[0–3], respectively; *p* < 0.001, *p* < 0.001, and *p* = 0.001, respectively; Bonferroni corrected *p* < 0.001, *p* < 0.001, and *p* = 0.002, respectively); however, the mean difference in NRS pain scores between the groups was small (−1 to −1, −1 to 0). The median NRS score at 6 h post-operation was not significantly different between the TRA and ROP groups (*p* = 0.651). The incidence of inadequate analgesia at 48 h post-operation in the TRA group (7.6% [31/410]) was not statistically different from that in the HYD (10.2% [42/410]; OR 1.40, 95% CI 0.86~2.27, *p* = 0.177) and the ROP groups (9.8% [40/410]; OR 1.32, 95% CI 0.81~2.16, *p* = 0.264), respectively. No significant difference was found between the TRA and HYD groups (*p* = 0.576) in the average of Ramsay sedation score after operation, as in the TRA and ROP groups (*p* = 0.795).

To further explore the risk factors for PPD in women who underwent cesarean delivery, we conducted an exploratory analysis using stepwise binary logistic regression (Table 4). Higher preoperative EPDS scores and longer lactation onset times were correlated with an increased risk of PPD at 4 weeks after cesarean delivery (OR 1.36, 95% CI 1.27–1.45, *p* < 0.001; OR 1.03, 95% CI 1.01–1.04, *p* = 0.001). History of dysmenorrhea was significantly correlated with the occurrence of PPD (OR 2.54, 95% CI 1.39–4.63, *p* = 0.002). A good marital relationship was associated with a lower risk of PPD (OR 0.37, 95% CI 0.18–0.76, *p* = 0.007). Taking tramadol PCIA as the reference group, hydromorphone PCIA and ropivacaine PCEA were significantly correlated with the occurrence of PPD (OR 2.10, 95% CI 1.10–4.11; OR 2.24, 95% CI 1.14–4.40, *p* = 0.004).

**Table 4:** Stepwise binary logistic-regression models for selecting potential risk factors for PPD in 4 weeks after cesarean.

**Models**	**Parameter**	**b**	**SE**	**Waldx^**2**^**	***P*-Value**	**OR (95% CI)**
Model 1	Constant	−4.58	0.34	183.38	<0.001	0.01
	Preoperative EPDS	0.28	0.03	79.05	<0.001	1.32 (1.24–1.41)
Model 2	Constant	−5.37	0.45	141.75	<0.001	0.005
	Preoperative EPDS	0.29	0.03	79.12	<0.001	1.33 (1.25–1.42)
	Lactation onset time	0.02	0.008	9.52	0.002	1.02 (1.01–1.04)
Model 3	Constant	−5.65	0.48	141.06	<0.001	0.004
	Preoperative EPDS	0.29	0.03	77.53	<0.001	1.34 (1.25–1.43)
	Lactation onset time	0.03	0.008	11.07	0.001	1.03 (1.01–1.04)
	Dysmenorrhea	0.87	0.30	8.43	0.004	2.39 (1.33–4.32)
Model 4	Constant	−2.78	1.08	6.65	0.01	0.06
	Preoperative EPDS	0.29	0.03	77.52	<0.001	1.34 (1.26–1.43)
	Lactation onset time	0.03	0.008	12.34	<0.001	1.03 (1.01–1.04)
	Dysmenorrhea	0.91	0.31	8.95	0.003	2.49 (1.37–4.53)
	Marital relationship	−1.02	0.36	8.00	0.005	0.36 (0.18–0.73)
Model 5	Constant	−3.48	1.13	9.54	0.002	0.03
	Preoperative EPDS	0.30	0.03	79.97	<0.001	1.36 (1.27–1.45)
	Lactation onset time	0.03	0.008	11.76	0.001	1.03 (1.01–1.04)
	Dysmenorrhea	0.93	0.31	9.17	0.002	2.54 (1.39–4.63)
	Marital relationship	−0.99	0.37	7.30	0.007	0.37 (0.18–0.76)
	Tramadol PCIA			6.43	0.04	
	Hydromorphone PCIA	0.74	0.34	4.65	0.03	2.10 (1.07–4.11)
	Ropivacaine PCEA	0.81	0.35	5.45	0.02	2.24 (1.14–4.40)

*Preoperative EPDS and GAD-7 were continuous variables; dysmenorrhea was recorded as Yes or No; marital relationship was divided into three levels (bad, general, and good); tramadol PCIA, hydromorphone PCIA, and ropivacaine PCEA were set as dummy variables, taking tramadol PCIA as the reference group. CI, Confidence interval; EPDS, Edinburgh Postpartum Depression Scale; OR, Odds ratio; PCEA, Patient-controlled epidural analgesia; PCIA, Patient-controlled intravenous analgesia*.

The incidence of related AEs is shown in Table 5. In addition to dizziness, the incidence of nausea and vomiting, pruritus, and headache were not significantly different among the three groups (*p* = 0.022, *p* = 0.072, *p* = 0.803, and *p* = 0.803, respectively). The incidence of dizziness in the TRA group (2.2% [9/410] was significantly higher than that in the ROP group (0.2% [1/410], OR 0.11, 95% CI 0.01–0.86, Bonferroni corrected *p* = 0.022), but not statistically different from that in the HYD group (1% [4/410], OR 0.44, 95% CI 0.13–1.44, *p* = 0.162). At 24 h postoperatively, 48 patients in the ROP group and no patients in the TRA and HYD groups reported hypoesthesia. Reported AEs in mothers were mostly mild and subsided soon after the end of the infusion. No cases of abnormal sleepiness, difficulty in breastfeeding, and breathing problems for infants were reported during the study. No of the patients discontinued treatment due to AEs.

**Table 5:** Adverse events in the three postoperative analgesia groups.

	**Tramadol PCIA** ***n* = 410**	**Hydromorphone PCIA** ***n* = 410**	**Ropivacaine PCEA** ***n* = 410**	***P-*value**
Nausea and vomiting	34 (8.3%)	26 (6.3%)	18 (4.4%)	0.072
Dizziness	9 (2.2%)	4 (1%)	1 (<1%)	**0.022**
Pruritus	1 (<1%)	2 (<1%)	2 (<1%)	0.803
Headache	1 (<1%)	2 (<1%)	2 (<1%)	0.803

*Data are described as number (percentage). PCEA, Patient-controlled epidural analgesia; PCIA, Patient-controlled intravenous analgesia. The bold values mean the significant differences (p < 0.05)*.

## Discussion

In this RCT involving women who underwent eCS, tramadol PCIA led to a lower incidence of PPD at 4 weeks postpartum in high-risk women and better overall QoR and sleep quality than one or both of the control groups.

Evidence shows that early intervention is vital for the prevention of PPD (43). Our perioperative tramadol PCIA strategy demonstrates the dual benefits of reducing the occurrence of PPD while relieving postoperative pain for the high risk of the PPD population. It is known that tramadol is relatively safe for breastfeeding. These results suggest that tramadol PCIA could be a rational strategy for early intervention of PPD following planned CS. Although dexmedetomidine PCIA after CS reduces the incidence of PPD and pain, it is not clear how much of the medication enters the breastmilk (44). Other medications, such as brexanolone, a novel antidepressant, and sertraline, a traditional first-line antidepressant, have demonstrated improvements in PPD (14, 45). However, these do not offer postoperative analgesia, and are aimed at intervention after the onset of PPD, which requires long treatment cycles with limited curative effect (14, 45).

Tramadol is a weak opioid agonist with selectivity for the μ-receptor. It also inhibits serotonin and norepinephrine uptake like tricyclic antidepressive agents (TCAs) and reduces alpha-2-adrenergic receptors (46) that are related to depression (47, 48). Our study included a primary control group (hydromorphone PCIA, commonly used after CS with a pure μ opioid receptor agonist). Our findings suggest tramadol may exerts its antidepressant effects by inhibiting serotonin and norepinephrine uptake and/or downregulating the alpha-2-adrenergic receptors. In addition, pain and depression share common pathways in the brain (49, 50). The antidepressant effects of tramadol may be secondary to pain relief. The TRA group had similar pain control as the HYD group during postoperative 48 h, but slightly better pain control than the ROP group at postoperative 12, 24, and 48 h. The incidence of inadequate analgesia at 48 h post-operation was comparable between the TRA and control groups. A study has reported that the optimal concentration of ropivacaine when used alone for PCEA is 2 mg/ml (51). However, a higher incidence (20%) of numbness in both lower limbs was observed in our previous clinical observations, which prompted us to change the concentration of ropivacaine to 1.5 mg/ml. This may have contributed to the insufficient analgesia in the ROP group.

The QoR at 48 h post-operation was significantly better in the TRA group than that in the control group. QoR-15 is an easy-to-use scoring system that assesses the quality of postoperative recovery from the patient's perspective, including physical and mental well-being. It has good validity, reliability and reproducibility in patients after surgery with anesthesia (38, 39). Therefore, the results may indicate that the postoperative tramadol scheme improved the physical and mental well-being of women who underwent CS. In addition, the TRA group had sleep quality comparable to that of the HYD group on day 0 and day 1 post-operation, but significantly better sleep quality than the ROP group. This is consistent with earlier studies showing that opioids can improve sleep quality while relieving pain (52, 53). Good sleep quality can reduce the risk of depression (54–57). Thus, the antidepressant effect of TRA may be through not only the improvement of sleep and pain compared to HYD, but also through a comprehensive approach.

The incidence of PPD in the control group declined to a level comparable to that in the TRA group at 3 months post-operation. Further, evaluation showed similar remission rates and proportion of new PPD cases in the period from 4 weeks to 3 months post-operation in all groups. It has been shown that high levels of depressive symptoms remit over the perinatal period in most women (58), similar to our results. Taken together, the perioperative tramadol PCIA strategy may ameliorate PPD in advance.

No unexpected tolerability concerns were noted in the TRA group. AEs were generally manageable and subsided within a few h after the end of infusion (18). Considering the low potential of tramadol for addiction (59, 60) and the technique and relatively short duration of tramadol administration in this study, the risk of developing tramadol dependence is likely to be low.

Our study suggests that the tramadol PCIA strategy is a valuable perioperative treatment option for early intervention of PPD following eCS, especially in the high-risk groups. It also relieves postoperative pain. These dual effects remove the burden of additional time, cost and side effects associated with the use of a separate antidepressant medication and psychological therapy. Moreover, depression is underdiagnosed in pregnant women compared with non-pregnant women (61), particularly for PPD (62, 63), due to insufficient attention from patients and perioperative doctors. The perioperative tramadol strategy can help perioperative physicians to reduce the risk of PPD in high-risk women.

## Limitations

First, to survey the effects of pain relief on depression, the study included a second control group (ropivacaine PCEA). An epidural infusion of ropivacaine and fentanyl is considered more effective than epidural ropivacaine alone for relieving pain after major abdominal surgery (51); using ropivacaine alone in our study may not have optimized postoperative analgesia. However, a comparison between the two PCIA groups was reasonable. Second, only patients who underwent CS were evaluated. Our findings may not be generalizable to all pregnant women, such as those who undergo vaginal delivery. Third, we carried out tramadol PCIA for 48 h at a rate of 16 mg/h (64) and did not explore the effects of other doses or durations on the primary outcome. Fourth, although tramadol is unlikely to adversely affect nursing infant (65) and its related effect is currently poorly understood (no case was reported in our study, too), tramadol is metabolized by P450 iso enzymes CYP2D6 which, in patients who are ultra-rapid metabolizers, may convert tramadol to M1 at high and unsafe levels in both maternal blood and breast milk potentially (66). This problem deserves a specialized detailed scheme to be investigated in the future. Fifth, although, we have included a number of factors in the analysis of risk factors for PPD, other risk factors may exist. Finally, the assessment of sleep quality in the current study was self-reported, which is subject to poor recall and bias.

## Conclusion

Tramadol PCIA in high-risk women who undergo eCS reduces the risk of PPD in the early postpartum period, relieves pain, and improves patients' quality of life and sleep quality post-operation. Future studies are required to fully elucidate the precise mechanisms underlying the antidepressant effects of tramadol. More work is also be needed to determine the optimal dose and duration of tramadol PCIA for reducing PPD and explore its efficacy in women undergoing vaginal delivery.

## Prior Presentations

The research findings in this manuscript have not been Published previously nor presented in any abstract or poster.

## Data Availability Statement

The original contributions presented in the study are included in the article/Supplementary Material, further inquiries can be directed to the corresponding author/s.

## Ethics Statement

The studies involving human participants were reviewed and approved by the Medical Ethics Committee of the Second Affiliated Hospital, Army Military Medical University. The patients/participants provided their written informed consent to participate in this study.

## Author Contributions

ZW wrote the paper. HL was involved in the study design, supervision of the data collection, reviewing of the report, quality checking, and critical revision. ZZ reviewed the paper. QD and GD performed the statistical analysis. QZ provided the training, counseling, and diagnosis of mood disorders. ZC provided assistance from obstetrics department. ZW, PZ, JP, LF, JD, GY, YW, JZ, DW, and YL contributed to study conduct and data collection. All authors contributed to the article and approved the submitted version.

## Conflict of Interest

The authors declare that the research was conducted in the absence of any commercial or financial relationships that could be construed as a potential conflict of interest.
