# Supplementary material for: A Patient-Controlled Intravenous Analgesia With Tramadol Ameliorates Postpartum Depression in High-Risk Woman After Cesarean Section: A Randomized Controlled Trial
Source: Front Med (Lausanne). 2021 May 27;8:679159. doi: 10.3389/fmed.2021.679159 (PMC8191376; doi:10.3389/fmed.2021.679159)
Supplement: Supplementary file 1 [file Table_1.DOCX]

Supplementary Material

# Supplementary Tables

**eTable 1. Demographics and perioperative clinical characteristics in women with preoperative EPDS ≥1**

|  | Tramadol  PCIA  n＝97 | Hydromorphone  PCIA  n＝86 | *P* value |  | Tramadol  PCIA  n＝97 | Ropivacaine  PCEA  n＝94 | *P* value |
| --- | --- | --- | --- | --- | --- | --- | --- |
| Age (years)^a^ | 30.00±4.42 | 30.93±4.31 | 0.15 |  | 30.00±4.42 | 30.49±4.43 | 0.45 |
| BMI (kg/m^2^)^a^ | 27.53±3.38 | 27.54±3.07 | 0.92 |  | 27.53±3.38 | 27.53±2.98 | 0.96 |
| Gestational weeks^b^ |  |  | 0.85 |  |  |  | 0.42 |
| Pre-term | 17（17.5%） | 14（16.3%） |  |  | 17（17.5%） | 12（12.8%） |  |
| Full-term | 80（82.5%） | 72（83.7%） |  |  | 80（82.5%） | 81（86.2%） |  |
| Post-term | 0（0%） | 0（0%） |  |  | 0（0%） | 1（1.1%） |  |
| Complication^b^ |  |  | 0.88 |  |  |  | 0.46 |
| No | 33（34.0%） | 28（32.6%） |  |  | 33（34.0%） | 37（39.4%） |  |
| Yes | 64（66.0%） | 58（67.4%） |  |  | 64（66.0%） | 57（60.6%） |  |
| Number of CSs^b^ |  |  | 0.12 |  |  |  | 0.44 |
| 0 | 31（32.0%） | 24（27.9%） |  |  | 31（32.0%） | 37（38.9%） |  |
| 1 | 58（59.8%） | 61（70.9%） |  |  | 58（59.8%） | 48（50.5%） |  |
| 2 | 7（7.2%） | 1（1.2%） |  |  | 7（7.2%） | 9（9.5%） |  |
| 3 | 1（1.0%） | 0（0.0%） |  |  | 1（1.0%） | 1（1.1%） |  |
| History of surgery (other than CS)^b^ | | | 0.73 |  |  |  | 0.92 |
| 0 | 70（72.2%） | 65（75.6%） |  |  | 70（72.2%） | 69（73.4%） |  |
| 1 | 24（24.7%） | 18（20.9%） |  |  | 24（24.7%） | 21（22.3%） |  |
| 2 | 3（3.1%） | 2（2.3%） |  |  | 3（3.1%） | 3（3.2%） |  |
| 3 | 0（0%） | 0（0.0%） |  |  | 0（0%） | 1（1.1%） |  |
| 4 | 0（0%） | 1（1.2%） |  |  | 0（0%） | 0（0%） |  |
| Sleep quality in the last week^b^ | | | 0.75 |  |  |  | 0.73 |
| Very poor to poor | 34（35.1%） | 29（33.7%） |  |  | 34（35.1%） | 31（33.0%） |  |
| General | 46（47.4%） | 38（44.2%） |  |  | 46（47.4%） | 42（44.7%） |  |
| Good to very good | 17（17.5%） | 19（22.1%） |  |  | 17（17.5%） | 21（22.3%） |  |
| Preoperative GAD-7 ^b^ | |  | 0.60 |  |  |  | 0.81 |
| ≤9 | 87（89.7%） | 80(93.0%) |  |  | 87（89.7%） | 86(91.5%) |  |
| ＞9 | 10（10.3%） | 6(7.0%) |  |  | 10（10.3%） | 8(8.5%) |  |
| Operation duration（mins）^a^ | 84.65±22.79 | 84.79±33.96 | 0.97 |  | 84.65±22.79 | 80.62±24.42 | 0.24 |
| Postpartum hemorrhage^b^ |  |  | 0.39 |  |  |  | 0.44 |
| No | 92（94.8%） | 78（90.7%） |  |  | 92（94.8%） | 92（97.9%） |  |
| Yes | 5（5.2%） | 8（9.3%） |  |  | 5（5.2%） | 2（2.1%） |  |
| PCIA consumption (mL)^c^ | 184(164, 229) | 185(150, 230) | 0.58 |  | 184(164, 229) | 185(148, 211) | 0.34 |
| Occupation^b^ |  |  | 0.46 |  |  |  | 0.75 |
| Peasant | 4（4.1%） | 4（4.7%） |  |  | 4（4.1%） | 4（4.3%） |  |
| Worker | 3（3.1%） | 7（8.1%） |  |  | 3（3.1%） | 2（2.1%） |  |
| Office clerk or civil servant | 36（37.1%） | 33（38.4%） |  |  | 36（37.1%） | 29（30.9%） |  |
| Other | 54（55.7%） | 42（48.8%） |  |  | 54（55.7%） | 59（62.8%） |  |
| Educational level^b^ |  |  | 0.05 |  |  |  | 0.26 |
| ≤9 years | 28（28.9%） | 12（14.0%） |  |  | 28（28.9%） | 19（20.2%） |  |
| 10~12 years | 19（19.6%） | 23（26.7%） |  |  | 19（19.6%） | 26（27.7%） |  |
| ＞12 years | 50（51.5%） | 51（59.3%） |  |  | 50（51.5%） | 49（52.1%） |  |
| Spouse’s occupation^b^ |  |  | 0.33 |  |  |  | 0.64 |
| Peasant | 5(5%) | 2(2%) |  |  | 5(5%) | 3(3%) |  |
| Worker | 6(6%) | 11(13%) |  |  | 6(6%) | 7(7%) |  |
| Soldier | 2(2%) | 0(0%) |  |  | 2(2%) | 2(2%) |  |
| Office clerk or civil servant | 32(33%) | 29(34%) |  |  | 32(33%) | 23(24%) |  |
| Other | 52(54%) | 44(51%) |  |  | 52(54%) | 59(63%) |  |
| Spouse’s educational level^b^ |  |  | 0.23 |  |  |  | 0.83 |
| ≤9 years | 20(21%) | 14(16%) |  |  | 20(21%) | 17(18%) |  |
| 10~12 years | 34(35%) | 23(27%) |  |  | 34(35%) | 31(33%) |  |
| ＞12 years | 43(44%) | 49(57%) |  |  | 43(44%) | 46(49%) |  |
| Marital status ^b^ |  |  | 0.47 |  |  |  | 0.49 |
| Married | 97（100%） | 85（98.8%） |  |  | 97（100%） | 93（98.9%） |  |
| Unmarried | 0（0.0%） | 1（1.2%） |  |  | 0（0.0%） | 0（0%） |  |
| Divorced | 0（0.0%） | 0（0%） |  |  | 0（0.0%） | 1（1.1%） |  |
| Monthly household income (Yuan) ^b^ | |  | 0.59 |  |  |  | 0.82 |
| 0-1500 | 0（0%） | 1（1.2%） |  |  | 0（0%） | 1（1.1%） |  |
| 1500-4500 | 14（14.4%） | 9（10.5%） |  |  | 14（14.4%） | 12（12.8%） |  |
| 4500-9000 | 56（57.7%） | 48（55.8%） |  |  | 56（57.7%） | 51（54.3%） |  |
| 9000-35000 | 27（27.8%） | 28（32.6%） |  |  | 27（27.8%） | 29（30.9%） |  |
| ＞35000 | 0（0%） | 0（0%） |  |  | 0（0%） | 1（1.1%） |  |
| Marital relationship^b^ |  |  | 0.84 |  |  |  | 0.89 |
| Bad | 1（1.0%） | 1（1.2%） |  |  | 1（1.0%） | 2（2.1%） |  |
| General | 4（4.1%） | 2（2.3%） |  |  | 4（4.1%） | 3（3.2%） |  |
| Good | 92（94.8%） | 83（96.5%） |  |  | 92（94.8%） | 89（94.7%） |  |

Data are described as ^a^mean±standard deviation, ^b^number (percentage) or ^c^median with Iinterquartile range.

^a^ Analyzed by the Independent-Samples T Test

^b^ Analyzed by the Chi-square test

^c^ Analyzed by the Mann-Whitney U test

BMI = Body mass index, EPDS = Edinburgh Postpartum Depression Scale, GAD = Generalized Anxiety Disorder, PCEA = Patient-controlled epidural analgesia, PCIA = Patient-controlled intravenous analgesia, CS = cesarean section
